# Supplementary material for: Non-specific effects of Pneumococcal and Haemophilus vaccines in children aged 5 years and under: a systematic review
Source: BMJ Open. 2023 Dec 14;13(12):e077717. doi: 10.1136/bmjopen-2023-077717 (PMC10729116; doi:10.1136/bmjopen-2023-077717)
Supplement: Author's manuscript [file bmjopen-2023-077717.draft_revisions.pdf]

BMJ Open is committed to open peer review. As part of this commitment we make the peer review history of every article we publish publicly available.

When an article is published we post the peer reviewers' comments and the authors' responses online. We also post the versions of the paper that were used during peer review. These are the versions that the peer review comments apply to.

The versions of the paper that follow are the versions that were submitted during the peer review process. They are not the versions of record or the final published versions. They should not be cited or distributed as the published version of this manuscript.

BMJ Open is an open access journal and the full, final, typeset and author-corrected version of record of the manuscript is available on our site with no access controls, subscription charges or pay-per-view fees (http://bmjopen.bmj.com).

If you have any questions on BMJ Open's open peer review process please email info.bmjopen@bmj.com

BMJ Open

A Systematic Review of Non-Specific Effects of Pneumococcal and Haemophilus Vaccines in Children Aged Five Years and Under.

Journal:	*BMJ Open*
Manuscript ID	bmjopen-2023-077717
Article Type:	Original research
Date Submitted by the Author:	13-Jul-2023
Complete List of Authors:	Geraghty, Keith; University of Limerick, School of Medicine<br>Rooney, Darragh; University of Limerick, School of Medicine<br>Watson, Chris; Queen's University Belfast Wellcome-Wolfson Institute for Experimental Medicine<br>Ledwidge, Mark; University College Dublin College of Health Sciences, gHealth Research Institute<br>Glynn, Liam; University of Limerick, School of Medicine<br>Gallagher, Joe; Irish College of General Practitioners, Global Health
Keywords:	Public health < INFECTIOUS DISEASES, Community child health < PAEDIATRICS, Paediatric infectious disease & immunisation < PAEDIATRICS, VIROLOGY

SCHOLARONE™
Manuscripts

I, the Submitting Author has the right to grant and does grant on behalf of all authors of the Work (as defined in the below author licence), an exclusive licence and/or a non-exclusive licence for contributions from authors who are: i) UK Crown employees; ii) where BMJ has agreed a CC-BY licence shall apply, and/or iii) in accordance with the terms applicable for US Federal Government officers or employees acting as part of their official duties; on a worldwide, perpetual, irrevocable, royalty-free basis to BMJ Publishing Group Ltd ("BMJ") its licensees and where the relevant Journal is co-owned by BMJ to the co-owners of the Journal, to publish the Work in this journal and any other BMJ products and to exploit all rights, as set out in our licence.

The Submitting Author accepts and understands that any supply made under these terms is made by BMJ to the Submitting Author unless you are acting as an employee on behalf of your employer or a postgraduate student of an affiliated institution which is paying any applicable article publishing charge ("APC") for Open Access articles. Where the Submitting Author wishes to make the Work available on an Open Access basis (and intends to pay the relevant APC), the terms of reuse of such Open Access shall be governed by a Creative Commons licence – details of these licences and which Creative Commons licence will apply to this Work are set out in our licence referred to above.

Other than as permitted in any relevant BMJ Author's Self Archiving Policies, I confirm this Work has not been accepted for publication elsewhere, is not being considered for publication elsewhere and does not duplicate material already published. I confirm all authors consent to publication of this Work and authorise the granting of this licence.

For peer review only

A Systematic Review of Non-Specific Effects of Pneumococcal and Haemophilus Vaccines in Children Aged Five Years and Under.

Authors

K Geraghty (School of Medicine UL);

Corresponding Author

School of Medicine,

University of Limerick, Limerick, Ireland

gerokeith@gmail.com

D Rooney (School of Medicine UL);

C Watson (School of Medicine UCD);

M Ledwidge (School of Medicine UCD);

L Glynn (School of Medicine UL);

J Gallagher (School of Medicine UCD)

For peer review only

For peer review only - http://bmjopen.bmj.com/site/about/guidelines.xhtml

ABSTRACT

Objective To determine the evidence for non-specific effects of the pneumococcal and haemophilus influenza vaccine in children aged five years and under.

Data sources A key word literature search of MEDLINE, Embase, The Cochrane Central Register of Controlled Trials, the European Union Clinical Trials Register, and clinicaltrials.gov up to June 2023.

Study eligibility criteria Randomised controlled trials, quasi-RCT or cohort studies.

Participants Children aged five or under.

Study appraisal and synthesis methods Studies were independently screened by two reviewers, with a third where disagreement arose. Risk of bias assessment was performed by one reviewer and confirmed by a second. Results were tabulated and a narrative description performed.

Results Four articles were identified and included in this review. We found a reduction in hospitalisations from influenza A (44%), Pulmonary TB (42%), metapneumovirus (45%), PIV Type 1-3 (44%), along with reductions in mortality associated with pneumococcal vaccine. No data on the haemophilus vaccine was found.

For peer review only

Conclusions and implications In this systematic review, we demonstrate that there is a reduction in particular viral infections in children aged five years and under who received the PCV9 vaccine which differ from those for which the vaccine was designed to protect against.

Review Registration The systematic review protocol was registered with the PROSPERO database (CRD42020146640)

For peer review only

STRENGTHS AND LIMITATIONS OF THIS STUDY

1. This systematic review explored the potential non-specific effects of two commonly administered vaccines in children aged five and under.
2. This systematic review was based on the systematic literature search following Preferred Reporting Items for Systematic review and Meta-Analysis.
3. Three of the articles reviewed were a post-hoc analysis of a phase III randomised, double-blind placebo-controlled PCV9 trial, using the same dataset.
4. No studies were found which explored the haemophilus vaccine in isolation.

For peer review only

For peer review only - http://bmjopen.bmj.com/site/about/guidelines.xhtml

INTRODUCTION

The beneficial effects of vaccination in reducing morbidity and mortality from the illnesses they are designed to prevent is widely reported with estimates that vaccines prevent approximately 6 million deaths worldwide annually [1, 2]. Vaccination has attempted to reduce morbidity and mortality from major diseases such as diphtheria, tetanus, yellow fever, pertussis, haemophilus influenza type b disease, measles, mumps, rubella, typhoid and rabies, with the successful eradication of smallpox and near-complete eradication of poliomyelitis[3].

Pneumonia remains a major cause of morbidity and mortality worldwide. The World Health Organisation (WHO) estimates there to be 156 million cases of pneumonia each year in children younger than five years, with as many as 20 million requiring hospital admission, accounting for an estimated 0.935 million deaths every year[4, 5]. However, the number of pneumonia deaths in young children has almost halved between 2000 and 2015[6]. This is likely attributable to improved provision of primary care through the Integrated Management of Childhood Illness programme, and increasing use of universal vaccination, including haemophilus influenza type B (Hib) and pneumococcal vaccines[7].

Vaccination with the Hib and pneumococcal conjugate vaccines protect children from invasive disease caused by these organisms. Routine vaccination has been shown to dramatically decrease the incidence of invasive Hib disease in children leading to a reduction in the number of hospital admissions[8, 9]. A study by Cowgill *et al.*[9] reported a reduction of the incidence of invasive Hib disease to 12% of its baseline level three years after the vaccine was

For peer review only

introduced. The 7-valent pneumococcal conjugate vaccine (PCV7) has been shown to reduce incidence rates of invasive pneumococcal disease by as much as 75%[10]. The introduction of the 13-valent pneumococcal conjugate vaccine (PCV13), replacing PCV7 in 2010, is likely to further reduce the incidence of pneumonia due to enhanced coverage of serotypes responsible for the majority of pneumococcal pneumonia cases in children worldwide[11]. GAVI, the Vaccine Alliance – previously the Global Alliance for Vaccines and Immunization – has been pivotal in the introduction of vaccination protocols enabling an estimated reduction of between 6 to 7.5 million cases of pneumonia and the avoidance of between 230,000 to 290,000 deaths since its introduction of its pneumococcal vaccine programme in 2007[12].

Furthermore, there is a body of evidence that certain vaccines yield beneficial effects not just limited to the illnesses they are designed to prevent. These effects are commonly termed "non-specific" effects. Vaccination with measles-containing vaccines, Bacille Calmette-Guérin (BCG), and oral polio vaccine (OPV) have all been shown to reduce both hospitalisation rates and mortality in children with further research required to better understand the impact on mortality[13-15]. Non-specific beneficial effects of vaccination in pneumonia may be due to bacteria and viruses acting as co-pathogens in the aetiology of pneumonia[16]. Evidence also indicates that respiratory viruses contribute to bacterial infections, often leading to bacterial superinfections[16]. Non-specific beneficial effects of vaccination may be due to the results of altered immune system memory, leading to a reduction in all-cause mortality[17, 18]. One study proposes that live vaccines can induce innate immune training, producing pro-inflammatory responses to unrelated antigens [19]. However, there is contradictory evidence, with one systematic review demonstrating that receipt of the diphtheria pertussis tetanus (DPT) vaccine was shown to be associated with an increase in all-cause mortality. This should be

For peer review only

For peer review only - http://bmjopen.bmj.com/site/about/guidelines.xhtml

interpreted with caution as all ten studies within the analysis were observational and classed as having a “high risk of bias.”[19, 20]

In recent years, researchers have offered a compelling argument for a radical change in the current vaccine paradigm [22], and newly suggested principles could mean a drastic overhaul in how countries administer their vaccines programmes. For example, it is suggested that live vaccines yield non-specific effects which are beneficial [23-27], while non-live vaccines may have detrimental non-specific effects, especially for females [28, 29]. In addition, it is proposed that non-specific effects of vaccines are determined by their most recent vaccine [30].

Further work is required to determine the presence or absence of non-specific effects of newer vaccines such as pneumococcus and haemophilus influenza which were more recently added to schedules internationally. In this study, we aim to determine if the administration of pneumococcal and/or Hib vaccines in infancy is associated with an effect on survival or hospitalisations from infections other than those conditions that the vaccine is designed to prevent in children up to five years of age. Furthermore, we aim to explore if the administration of pneumococcal and/or Hib vaccines in infancy are associated with an effect on severity of the illness as defined by the authors, or hospitalisations for infections other than those conditions that the vaccine is designed to prevent in children up to five years of age.

For peer review only - http://bmjopen.bmj.com/site/about/guidelines.xhtml

METHOLODOLOGY

The systematic review protocol was registered with the PROSPERO database (CRD42020146640), which guided the authors in conducting this review. The preferred reporting items for systematic reviews and meta-analyses (PRISMA) statement[31] was used to guide the reporting and conduct of the review.

Eligibility Criteria

To be eligible for inclusion, studies had to meet certain predetermined criteria outlined in the review protocol. The population of interest was children aged five and under. Haemophilus vaccines along with any formulation of the pneumococcal vaccine, including PCV7, PCV13, and PCV23 were included. Studies reporting survival, all-cause mortality, or deaths from infections other than those conditions that the vaccine was designed to prevent, or studies reporting deaths from all causes (e.g., all-cause mortality, child survival) or severity as defined by the authors (e.g., hospitalisation) were eligible for inclusion. Only randomised controlled trials (RCTs), quasi-RCTs or cohort studies written in English were included in this review.

Exclusion Criteria

Ecological studies, uncontrolled studies (i.e., case reports and case series studies), "case-only" studies, and self-controlled case series studies were excluded from this review, as these studies provide less reliable data for assessing non-specific effects of vaccine on hospitalisations and mortality. Additionally, animal and laboratory studies were excluded. Studies which included adults or children over the age of five, or those which did not report children under the age of five separately, were not included in this review.

For peer review only

Data Management

Results from the literature search were directly imported into the online Rayyan software package[32]. This was used for screening through the citations and abstracts from the electronic databases, as well as the full-text articles indicated via the search. Two reviewers independently screened the literature search results for inclusion. They then independently reviewed the full text of potentially relevant articles and screened them to determine inclusion using the same inclusion and exclusion criteria. In cases of disagreement a third reviewer acted as arbitrator.

Search Strategy

The search strategy was jointly developed and tested during the protocol phase and was subsequently finalised by reviewers prior to commencing the search. Medical subject headings and text words related to children, pneumococcal and haemophilus influenza vaccination were used. Electronic databases MEDLINE (OVID interface, 1948 onwards)[33], EMBASE (OVID interface, 1980 onwards)[34] and Pubmed were each searched using the predetermined search terms. The Cochrane Central Register of Controlled Trials (Wiley interface, current issue)[35] and European Union Clinical Trials Register (EURACT) were searched to supplement the search. All reference lists of included studies were scrutinised and reviewed to further identify relevant reviews not found during the database search. Finally, a bibliography of included articles was circulated to experts in the field to ensure literature saturation.

For peer review only - http://bmjopen.bmj.com/site/about/guidelines.xhtml

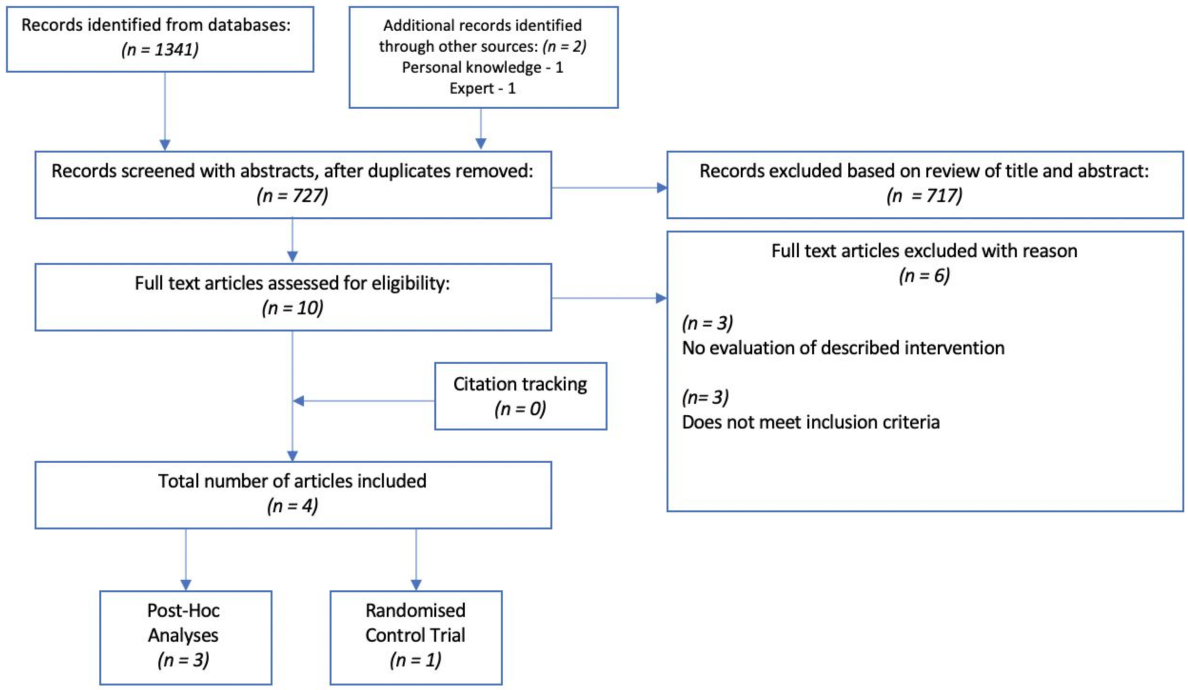

PRISMA Flow Diagram

Data Collection Process

To ensure data accuracy, two reviewers independently abstracted the data using a standardised data abstraction form. This form was developed during the protocol phase and was trialled and refined prior to commencement of the review.

Risk of Bias Assessment

We used Cochrane tools for assessing risk of bias in clinical trials (RoB2)[36] for randomised studies. Any potential confounders were considered during analysis of individual studies, and we determined if these were reported. Confounders included age and gender distribution, health status of the children (including nutritional status and birth weight), and socioeconomic status (including location of the study, poverty, education, and hygiene conditions). We also assessed as to whether any other interventions were undertaken

For peer review only - http://bmjopen.bmj.com/site/about/guidelines.xhtml

including malaria interventions, de-worming, co-administration of vitamin A or zinc or other micronutrient supplements, breast feeding, hygiene programmes, water programmes and other vaccinations that may have been reported in the studies.

Data Synthesis

To effectively synthesise the data obtained, data was grouped together and tabulated. Textual descriptions were provided. Studies were contrasted, and a narrative on reported outcomes, effectiveness of studies, and design strengths and weaknesses were presented.

Patient and Public Involvement

As this is a systematic review of previously published research, the authors have no direct or indirect involvement with patients or the public.

Ethical Approval

This review does not require ethical approval as authors have retrieved and synthesised previously published literature.

For peer review only

For peer review only - http://bmjopen.bmj.com/site/about/guidelines.xhtml

RESULTS

We retrieved 1,341 articles from MEDLINE, EMBASE, and PubMed electronic databases using our search strategy. Rayyan software automatically removed duplicate records, and suggested articles which were similar in title or author, allowing manual removal of any duplicates not successfully detected by the software package. We had prior knowledge of one article which met our inclusion criteria, which was therefore included in the screening process. One further article was suggested by an expert in the field, and this was also included. After duplicates were removed, 727 records remained and were subsequently screened by two reviewers independently. Of these, 717 were excluded based on screening abstracts and titles. Ten full-text articles were assessed for eligibility, using the eligibility form. Six of these were excluded as they did not meet inclusion criteria *(n = 3*), or they did not evaluate the described intervention *(n = 3).* Scanning references from the remaining articles did not reveal any new articles. Therefore, four studies were included in our systematic review. Three studies were post-hoc analyses of the same randomised control trial dataset. One study was a randomised control study.

All four studies were associated with the use of the 9-valent pneumococcal conjugate vaccine. No study involving the use of the haemophilus influenza vaccine was identified.

Considering the use of common datasets, 40,100 children under the age of five were involved in these studies. Summaries of the main findings of each study are outlined in *Table 1.* Results of these studies are summarised in *Table 2.*

For peer review only - http://bmjopen.bmj.com/site/about/guidelines.xhtml

Table 1: Study Characteristics

Authors	Study Type	Country	Population	Subpopulation	Vaccine	Control	Other Vaccines concurrently administered
Moore DP, Klugman KP, Madhi SA (2010)[37]	Post-hoc analysis of a phase III randomised, double-blind placebo-controlled PCV9 trial	South Africa	39,836 children, aged 84 days and under	HIV-infected and HIV-uninfected	9-valent pneu- mococcal vaccine or a placebo<br><br>[2 µg of capsular polysac-charide (serotypes 1, 4, 5, 9V, 14, 19F, and 23F), 4 µg of serotype 6B, and 2 µg of oligosaccharide 18C]	Haemophilus influenzae type b conjugate vaccine	Diphtheria, tetanus, and whole-cell pertussis (DTwP, Aventis Pasteur); hepatitis B (Hepaccine-B, Cheil Sugar Organization); and oral live, trivalent poliovirus types 1, 2, and 3 (Poli- oral, Biovac).
Madhi SA, Klugman KP, & The Vaccine Trialist Group (2004)[38]	Post-hoc analysis of a phase III randomised, double-blind placebo-controlled PCV9 trial	South Africa	39,836 children, aged 84 days and under	HIV-infected and HIV-uninfected	9-valent pneu- mococcal vaccine or a placebo<br><br>[2 µg of capsular polysac-charide (serotypes 1, 4, 5, 9V, 14, 19F, and 23F), 4 µg of serotype 6B, and 2 µg of oligosaccharide 18C]	Haemophilus influenzae type b conjugate vaccine	Diphtheria, tetanus, and whole-cell pertussis (DTwP, Aventis Pasteur); hepatitis B (Hepaccine-B, Cheil Sugar Organization); and oral live, trivalent poliovirus types 1, 2, and 3 (Poli- oral, Biovac).
Madhi, S.A., et al. (2006)[39]	Post-hoc analysis of a phase III randomised, double-blind placebo-controlled trial	South Africa	39,836 children (aged 60-84 days)	HIV-infected and HIV-uninfected	9-valent pneu- mococcal vaccine or a placebo<br><br>[2 µg of capsular polysac-charide (serotypes 1, 4, 5, 9V, 14, 19F, and 23F), 4 µg of serotype 6B, and 2 µg of oligosaccharide 18C]	Haemophilus influenzae type b conjugate vaccine	Diphtheria, tetanus, and whole-cell pertussis (DTwP, Aventis Pasteur); hepatitis B (Hepaccine-B, Cheil Sugar Organization); and oral live, trivalent poliovirus types 1, 2, and 3 (Poli- oral, Biovac).
Dagan R, Sikuler-Cohen M, Zamir O, Janco J, Givon-Lavi N, Fraser D (2001)[40]	Randomised, double-blind placebo-controlled PCV9 trial	Israel	264 children (aged 12-35 months)	-	9-valent pneumococcal conjugate vaccine<br><br>[2 µg of capsular polysac-charide (serotypes 1, 4, 5, 9V, 14, 18C, 19F and 23F), 4 µg of serotype 6B, coupled to diphtheria toxin CRM197]	Meningococcus group C conjugate vaccine	Not reported

For peer review only

Study	Subpopulation	Illness	Outcome Measured	Efficacy (95% CI)	*P*
Moore DP, Klugman KP, Madhi SA (2010)[37]	HIV-uninfected	First Episode Culture-Confirmed PTB	All-Cause Hospitalisations	35.3 (-38.1-69.7)	0.2562
		All categories of first episode PTB		0.0 (-40.9-29.1)	0.9983
		All culture-confirmed PTB		38.9 (-29.3-71.1)	0.1931
	HIV-infected	First Episode Culture-Confirmed PTB		47.3 (8.6-69.6)	0.0203
		All categories of first episode PTB		13.2 (-10.4-31.8)	0.2475
		All culture-confirmed PTB		43.6 (5.5-66.4)	0.0274
	Overall	First Episode Culture-Confirmed PTB		43.4 (11.5-63.8)	0.0114
		All categories of first episode PTB		8.8 (-11.7-25.5)	0.9983
		All culture-confirmed PTB		42.1 (11.2-62.3)	0.0113
Madhi SA, Klugman KP, & The Vaccine Trialist Group (2004)[38]	HIV-uninfected	Any Virus-Associated Pneumonia	All-Cause Hospitalisations	33 (15-48)	0.0008
		Influenza A		34 (-14-62)	0.1
		RSV		32 (6-50)	0.02
		PIV Types 1-3		41 (-10-68)	0.09
		Adenovirus		31 (-62-70)	0.4
	HIV-infected	Any Virus-Associated Pneumonia		0.2 (-14-47)	23
		Influenza A		57 (7-80)	0.03
		RSV		-30 (-140-31)	0.4
		PIV Types 1-3		50 (-17-78)	0.1
		Adenovirus		-150 (-1188-51)	0.3
	Overall	Any Virus-Associated Pneumonia		31 (15-43)	0.0004
		Influenza A		45 (14-64)	0.01
		RSV		22 (-3-41)	0.08
		PIV Types 1-3		44 (8-66)	0.02
		Adenovirus		7 (-94-55)	0.9
Madhi, S.A., et al. (2006)[39]	HIV-uninfected	**Human Metapneumovirus-Associated LRTI**	All-Cause Hospitalisations		
		<6.0 months		6 (-83-51)	0.87
		6.1-12 months		52 (7-75)	0.027
		12.1-24 months		33 (-17-62)	0.16
		>24 months		47 (-13-76)	0.09
	HIV-infected	<6.0 months		34 (-295-89)	0.65

For peer review only

	 <br><br><br>Overall	6.1-12 months<br>12.1-24 months<br>>24 months<br><br><6.0 months<br>6.1-12 months<br>12.1-24 months<br>>24 months		43 (-93-83)<br>58 (-64-89)<br>42 (-46-77)<br><br>14 (-59-53)<br>52 (14-73)<br>40 (-1-64)<br>45 (1-70)	0.36<br>0.20<br>0.24<br><br>0.63<br>0.012<br>0.053<br>0.04
Dagan R, Sikuler-Cohen M, Zamir O, Janco J, Givon-Lavi N, Fraser D (2001)[40]	-	**URI**<br><36 months<br>>36 months<br>Overall<br><br>**All-cause LRTI**<br><36 months<br>>36 months<br>Overall<br><br>**Otitis Media**<br><36 months<br>>36 months<br>Overall	Hospitalisations	 17 (0.7-0.99)<br>14 (0.73-1.02)<br>15 (0.76-0.96)<br><br><br>23 (0.62-0.95)<br>9 (0.73-1.12)<br>16 (0.72-0.98)<br><br><br>23 (0.58-1.03)<br>12 (0.65-1.20)<br>17 (0.67-1.02)	 0.036<br>0.082<br>0.009<br><br><br>0.015<br>0.371<br>0.024<br><br><br>0.075<br>0.425<br>0.078

Table 2: Results

For peer review only

Three of the studies[37-39], all of which undertook a *post-hoc* analysis of a prospective randomised, double-blind placebo-controlled PCV9 efficacy study demonstrated a reduction in hospitalisations from infections other than those that the pneumococcal conjugate vaccine was designed to prevent against. The trial, conducted in South Africa, enrolled 39,836 children between 1 March 1998 and October 2000. Subjects received either PCV9 or a control, and investigators and laboratory staff remained blinded to the randomisation throughout the surveillance phase. The mortality rate was reduced by 5 percent among all children (*p=0.58),* and by 6 percent among HIV-infected children *(p=0.63)* in this study.

Upon analysis of this dataset, Moore *et al.*[37] determined that there was a reduction in hospitalisation for culture-confirmed pulmonary tuberculosis (PTB) of 43.4% in the vaccine study group versus the control group (95% CI: 11.5-63.8; *p=0.0114.)* In HIV-infected children, the risk of being hospitalised with a lower respiratory tract infection (LRTI) was 47.3% lower (95% CI: 8.6-69.6; *p=0.0203*) in PCV9 recipients compared to the placebo recipients. Although not statistically significant, a similar trend was observed in children uninfected by HIV (relative risk reduction 35.3% [95% CI: -38.1-69.7]; *p*=0.2562). Where available, intention-to-treat (ITT) results are described as it reflects the practical clinical scenario and gives an unbiased estimate of treatment effect[41].

In their analysis of the same dataset, Madhi *et al.*[38] show that in all children (i.e. those with or without HIV infection), PCV9 reduced pneumonias associated with any of the identified viruses by 31% (95% CI: 15-43; *p=0.0004*). Furthermore, there were similar point estimates of efficacy with Influenza A (45%; 95 CI: 14-64; *p=0.01)*, parainfluenza virus type 1-3 (44%;

For peer review only - http://bmjopen.bmj.com/site/about/guidelines.xhtml

95% CI: 3-64%; *p=0.01)* and respiratory syncytial virus (22%; 95 CI: -3-41; *p=0.08*) in all children.

In a further study, Madhi *et al.*[39] demonstrated a reduction in incidence of human metapneumovirus in both HIV-infected and HIV-unaffected children who received the PCV9 vaccine, (reduction of 53% [95% CI: 3-77; *p=0.035*] vs 45% reduction [95% CI: 19-62; *p=0.002*], in the per protocol analysis, respectively). In this study, the authors also note a significant reduction in the incidence of clinical pneumonia among vaccine recipients overall (58; 95% CI 34-73; *p=0.0002*).

Dagan *et al*.[40] conducted a small randomised, double-blind, placebo-controlled study in Israel, whereby 264 toddlers received either PCV9, or a control (meningococcal conjugate vaccine). This study revealed a 23% reduction (95% CI: 0.62-0.95; *p=0.015*) in all-cause lower respiratory infection in children under 36 months compared with placebo recipients. A 17% reduction in upper respiratory tract infection was observed in the same age group (95% CI: 0.7-0.99; *p=0.036*). Although there was a similar trend in children >36 months, this was not statistically significant.

Each study was assessed for quality, and a risk of bias assessment was made using the RoB2[36] tool. All studies were deemed low risk of bias. We considered any potential confounders and based on the location of the trials, found the socioeconomic status of the population to be a potential confounder for both the South African and Israeli studies. Certain populations are more likely to live in overcrowded communities, potentially subjecting them to a higher burden of disease [42]. Furthermore, we felt socioeconomic status was important as it may

For peer review only - http://bmjopen.bmj.com/site/about/guidelines.xhtml

determine inequalities in healthcare access. In all papers, there was no discussion which raised possible implications for vaccinating children in areas where disease may be more prevalent.

For peer review only

DISCUSSION

Reports to date have determined the efficacy of PCV9 in the reduction of nasopharyngeal pneumococcal carriage[43], invasive infections[44] and mucosal infections[44]. In this systematic review, we demonstrate that there is a reduction in particular viral infections in children under the age of five who received the PCV9 vaccine, which differ from those for which the vaccine was designed to protect against.

Reduction in the incidence of hospitalisation from other diseases was reported most frequently. *Post-hoc* analyses from a phase III randomised control trial in South Africa reveal a reduction in culture-confirmed pulmonary TB[37], influenza A[38], parainfluenza type 1-3[38], and human metapneumovirus[39] in all children, irrespective of their HIV status. In a smaller RCT in Israel [40], a reduction in all-cause lower respiratory tract infections was determined in day-care centre attendees.

The authors of the phase III randomised control trial included analysis of HIV-infected versus non-infected children, which allowed post-hoc analysis of these subgroups. While the hospital reductions mentioned above include all children, the reduction of PTB in vaccinee recipients is only statistically significant in HIV-infected children, and overall[37]. Furthermore, the reduction of RSV was only significant in all-children, and those not infected with HIV[38]. All-cause mortality did not differ between groups in this trial.

For peer review only - http://bmjopen.bmj.com/site/about/guidelines.xhtml

While included trials have a relatively equal representation of both sexes in their population, there is no analysis of potential non-specific effects of the respective vaccines on males versus females. As described, several non-live vaccines, namely DPT [45], inactive polio vaccine [46], hepatitis B vaccine[47], measles and yellow fever vaccine[48] are associated with increased female mortality. Given the pneumococcal vaccine is inactivated, further work exploring the sex-differential is warranted.

Interestingly, the control used in the randomised control trial in South Africa was the Haemophilus influenzae type b conjugate vaccine, the second vaccine we sought to explore in this review. This was given so those children in the control group could infer some benefit. It is therefore likely that these subsets would receive some protection against haemophilus influenza. No study was identified which sought to explore non-specific effects in the Hib vaccine in isolation, and further research is required in this domain.

The South African trial reported on other vaccinations children received as part of their routine schedule, although did not outline the sequence in which these vaccines were administered. Unfortunately, this information was omitted from the Dagan study. Certain combinations of vaccines are associated with increased mortality (e.g., DTP and measles vaccine)[49], whereas others are associated with lower mortality (e.g. DTP and OPV)[50]. In addition, there is evidence that combining non-live and live vaccines can have a detrimental effect on mortality[51]. Therefore, further detail regarding the sequence of vaccines is crucial, as it may impact efficacy and mortality.

For peer review only - http://bmjopen.bmj.com/site/about/guidelines.xhtml

This study has several limitations. Most notably, three of the articles reviewed were a *post-hoc* analysis of the same dataset, which used the same population. Post-hoc analyses typically describes findings for which the parent study was not designed to explore. In addition, the high proportion of studies in this area which come from common data sets is a weakness of this review. As discussed, analysis to include sex-differential and sequencing of vaccines is missing from these studies.

The findings of this review do however add to the body of evidence that certain vaccines infer additional non-specific effects. The most widely reported of these vaccines are the BCG and oral polio vaccine[14, 15], with some currently hypothesising that the BCG vaccine may in fact offer protection against the SARS-CoV-2 virus[52].

Vaccination programmes have demonstrated wide-ranging benefits, however some of these are poorly defined. For public health officials and those deciding on health policy, there is a need to prioritise vaccines which have an identifiable and preventable disease burden. Consideration of those non-specific effects which may impact upon morbidity and mortality are important in deciding upon novel vaccination strategies.

For peer review only

CONCLUSION

This systematic review suggests that the 9-valent pneumococcal conjugate vaccine may confer protection against infections other than those for which the vaccine was designed to protect against. Further research is warranted to fully explore the presence or absence of non-specific effects on the pneumococcal vaccine and in particular, the haemophilus influenza vaccine.

For peer review only

For peer review only - http://bmjopen.bmj.com/site/about/guidelines.xhtml

Funding

This study was conducted with the financial support of the Bill and Melinda Gates Foundation (OPP113957) and Science Foundation Ireland (SFI) and the Department of Foreign Affairs (DFA) under the SDG Challenge Grant Number SFI/21/FIP/SDG/9948

Author Contributorship

Geraghty, K – acquisition of data; analysis and interpretation of data; drafting of manuscript, primary reviewer

Rooney, D – conception and design of study; protocol design and registration; third reviewer

Watson, C – reviewer of manuscript

Ledwidge, Mark T – reviewer of manuscript

Glynn, L – reviewer of manuscript

Gallagher, J – conception and design of study; protocol design and registration; critical revisions of manuscript; primary reviewer

Conflict of Interest

All authors declare that they have no conflicts of interest.

Data Sharing Statement

Data is available upon reasonable request.

For peer review only - http://bmjopen.bmj.com/site/about/guidelines.xhtml

REFERENCES

1. Andre, F.E., et al., *Vaccination greatly reduces disease, disability, death and inequity worldwide.* Bull World Health Organ, 2008. **86**(2): p. 140-6.
2. Ehreth, J., *The global value of vaccination.* Vaccine, 2003. **21**(7-8): p. 596-600.
3. Plotkin, S., *History of vaccination.* Proc Natl Acad Sci U S A, 2014. **111**(34): p. 12283-7.
4. Rudan, I., et al., *Epidemiology and etiology of childhood pneumonia.* Bull World Health Organ, 2008. **86**(5): p. 408-16.
5. Liu, L., et al., *Global, regional, and national causes of child mortality in 2000-13, with projections to inform post-2015 priorities: an updated systematic analysis.* Lancet, 2015. **385**(9966): p. 430-40.
6. McAllister, D.A., et al., *Global, regional, and national estimates of pneumonia morbidity and mortality in children younger than 5 years between 2000 and 2015: a systematic analysis.* Lancet Glob Health, 2019. **7**(1): p. e47-e57.
7. Gove, S., *Integrated management of childhood illness by outpatient health workers: technical basis and overview. The WHO Working Group on Guidelines for Integrated Management of the Sick Child.* Bull World Health Organ, 1997. **75 Suppl 1**(Suppl 1): p. 7-24.
8. *Progress toward elimination of Haemophilus influenzae type b invasive disease among infants and children--United States, 1998-2000.* MMWR Morb Mortal Wkly Rep, 2002. **51**(11): p. 234-7.
9. Cowgill, K.D., et al., *Effectiveness of Haemophilus influenzae type b Conjugate vaccine introduction into routine childhood immunization in Kenya.* Jama, 2006. **296**(6): p. 671-8.

For peer review only

10. *Direct and indirect effects of routine vaccination of children with 7-valent pneumococcal conjugate vaccine on incidence of invasive pneumococcal disease--United States, 1998-2003.* MMWR Morb Mortal Wkly Rep, 2005. **54**(36): p. 893-7.
11. Resti, M., et al., *Community-acquired bacteremic pneumococcal pneumonia in children: diagnosis and serotyping by real-time polymerase chain reaction using blood samples.* Clin Infect Dis, 2010. **51**(9): p. 1042-9.
12. The Vaccine Alliance. *GAVI*. 2020 [cited 2020 06/09/2020]; Available from: https://www.gavi.org/.
13. Aaby, P., et al., *Non-specific effects of standard measles vaccine at 4.5 and 9 months of age on childhood mortality: randomised controlled trial.* Bmj, 2010. **341**: p. c6495.
14. de Castro, M.J., J. Pardo-Seco, and F. Martinón-Torres, *Nonspecific (Heterologous) Protection of Neonatal BCG Vaccination Against Hospitalization Due to Respiratory Infection and Sepsis.* Clin Infect Dis, 2015. **60**(11): p. 1611-9.
15. Lund, N., et al., *The Effect of Oral Polio Vaccine at Birth on Infant Mortality: A Randomized Trial.* Clin Infect Dis, 2015. **61**(10): p. 1504-11.
16. Peltola, V.T. and J.A. McCullers, *Respiratory viruses predisposing to bacterial infections: role of neuraminidase.* Pediatr Infect Dis J, 2004. **23**(1 Suppl): p. S87-97.
17. Saeed, S., et al., *Epigenetic programming of monocyte-to-macrophage differentiation and trained innate immunity.* Science, 2014. **345**(6204): p. 1251086.
18. Benn, C.S., et al., *A small jab - a big effect: nonspecific immunomodulation by vaccines.* Trends Immunol, 2013. **34**(9): p. 431-9.
19. Kleinnijenhuis J, Quintin J, Preijers F, et al. *Bacille Calmette-Guerin induces NOD2-dependent nonspecific protection from reinfection via epigenetic reprogramming of monocytes.* Proc Natl Acad Sci U S A 2012; 109(43): 17537-42.

For peer review only - http://bmjopen.bmj.com/site/about/guidelines.xhtml

20. Yung, C.F., *Non-specific effects of childhood vaccines.* Bmj, 2016. **355**: p. i5434.

21. Higgins, J.P., et al., *Association of BCG, DTP, and measles containing vaccines with childhood mortality: systematic review.* Bmj, 2016. **355**: p. i5170.

22. Be Benn, C. S., Fisker, A. B., Rieckmann, A., Sørup, S., & Aaby, P. (2020). *Vaccinology: time to change the paradigm?.* The Lancet. Infectious diseases, 20(10), e274–e283nn, C. S., Fisker, A. B., Rieckmann, A., Sørup, S., & Aaby, P. (2020). Vaccinology: time to change the paradigm?. The Lancet. Infectious diseases, 20(10), e274–e283

23. Sorup S, Benn CS, Poulsen A, Krause TG, Aaby P, Ravn H. *Live vaccine against measles, mumps, and rubella and the risk of hospital admissions for nontargeted infections.* JAMA 2014; 311(8): 826-35.

24. La Torre G, Saulle R, Unim B, et al. *The effectiveness of measles-mumps-rubella (MMR) vaccination in the prevention of pediatric hospitalizations for targeted and untargeted infections: A retrospective cohort study.* Hum Vaccin Immunother 2017; 13(8): 1879-83.

25. Upfill-Brown A, Taniuchi M, Platts-Mills JA, et al. *Nonspecific Effects of Oral Polio Vaccine on Diarrheal Burden and Etiology Among Bangladeshi Infants*. Clin Infect Dis 2017; 65(3): 414-9.

26. Seppala E, Viskari H, Hoppu S, et al. *Viral interference induced by live attenuated virus vaccine (OPV) can prevent otitis media.* Vaccine 2011; 29(47): 8615-8.

27. Rieckmann A, Villumsen M, Sorup S, et al. *Vaccinations against smallpox and tuberculosis are associated with better long-term survival: a Danish case-cohort study 1971-2010*. Int J Epidemiol 2017; 46(2): 695-705.

28. Aaby P, Jensen H, Samb B, et al. *Differences in female-male mortality after high-titre measles vaccine and association with subsequent vaccination with diphtheria-tetanus-*

For peer review only - http://bmjopen.bmj.com/site/about/guidelines.xhtml

pertussis and inactivated poliovirus: reanalysis of West African studies. Lancet 2003; 361(9376): 2183-8.

29. Holt EA, Moulton LH, Siberry GK, Halsey NA. *Differential mortality by measles vaccine titer and sex*. J Infect Dis 1993; 168(5): 1087-96
30. Aaby P, Ravn H, Benn CS, et al. Randomized Trials Comparing Inactivated Vaccine After Medium- or High-titer Measles Vaccine With Standard Titer Measles Vaccine After Inactivated Vaccine: A Meta-analysis. Pediatr Infect Dis J 2016; 35(11): 1232-41.
31. Moher, D., et al., *Preferred reporting items for systematic review and meta-analysis protocols (PRISMA-P) 2015 statement.* Syst Rev, 2015. **4**(1): p. 1.
32. Ouzzani, M., et al., *Rayyan-a web and mobile app for systematic reviews.* Syst Rev, 2016. **5**(1): p. 210.
33. MEDLINE. *MEDLINE*. 2020; Available from: https://www.ncbi.nlm.nih.gov/pubmed.
34. EMBASE. *EMBASE*. 2020; Available from: https://www.embase.com/login.
35. Cochrane, *Cochrane Central Register of Controlled Trials*. 2020.
36. Sterne, J.A.C., et al., *RoB 2: a revised tool for assessing risk of bias in randomised trials.* Bmj, 2019. **366**: p. l4898.
37. Moore, D.P., K.P. Klugman, and S.A. Madhi, *Role of Streptococcus pneumoniae in hospitalization for acute community-acquired pneumonia associated with culture-confirmed Mycobacterium tuberculosis in children: a pneumococcal conjugate vaccine probe study.* Pediatr Infect Dis J, 2010. **29**(12): p. 1099-04.
38. Madhi, S.A. and K.P. Klugman, *A role for Streptococcus pneumoniae in virus-associated pneumonia.* Nat Med, 2004. **10**(8): p. 811-3.
39. Madhi, S.A., et al., *Pneumococcal coinfection with human metapneumovirus.* J Infect Dis, 2006. **193**(9): p. 1236-43.

For peer review only

40. Dagan, R., et al., *Effect of a conjugate pneumococcal vaccine on the occurrence of respiratory infections and antibiotic use in day-care center attendees.* Pediatr Infect Dis J, 2001. **20**(10): p. 951-8.

41. Heritier, S.R., V.J. Gebski, and A.C. Keech, *Inclusion of patients in clinical trial analysis: the intention-to-treat principle.* Med J Aust, 2003. **179**(8): p. 438-40

42. Public Health England. Beyond the data: Understanding the impact of COVID-19 on BAME groups. 2020

43. Mbelle, N., et al., *Immunogenicity and impact on nasopharyngeal carriage of a nonavalent pneumococcal conjugate vaccine.* J Infect Dis, 1999. **180**(4): p. 1171-6.

44. Black, S., et al., *Efficacy, safety and immunogenicity of heptavalent pneumococcal conjugate vaccine in children. Northern California Kaiser Permanente Vaccine Study Center Group.* Pediatr Infect Dis J, 2000. **19**(3): p. 187-95.

45. Aaby P, Mogensen SW, Rodrigues A, Benn CS. Evidence of Increase in Mortality After the Introduction of Diphtheria-Tetanus-Pertussis Vaccine to Children Aged 6-35 Months in Guinea-Bissau: A Time for Reflection? *Front Public Health* 2018; **6**(79): 79.

46. Aaby P, Garly ML, Nielsen J, et al. Increased female-male mortality ratio associated with inactivated polio and diphtheria-tetanus-pertussis vaccines: Observations from vaccination trials in Guinea-Bissau. *Pediatr Infect Dis J* 2007; **26**(3): 247-52.

47. Garly ML, Jensen H, Martins CL, et al. Hepatitis B vaccination associated with higher female than male mortality in Guinea-bissau: an observational study. *Pediatr Inf Dis J* 2004; **23**(12): 1086-92.

48. Fisker AB, Biering-Sorensen S, Lund N, et al. Contrasting female-male mortality ratios after routine vaccinations with pentavalent vaccine versus measles and yellow fever vaccine. A cohort study from urban Guinea-Bissau. *Vaccine* 2016; **34**(38): 4551-7.

For peer review only

For peer review only - http://bmjopen.bmj.com/site/about/guidelines.xhtml

49. Higgins JP, Soares-Weiser K, Lopez-Lopez JA, et al. *Association of BCG, DTP, and measles containing vaccines with childhood mortality: a sytematic review*. BMJ 2016; 355:i5170.

50. Mogensen SW, Andersen A, Rodrigues A, Benn CS, Aaby P. The Introduction of Diphtheria-Tetanus-Pertussis and Oral Polio Vaccine Among Young Infants in Urban African Community: A Natural Experiment. EBioMedicine 2017; 17: 192-8.

51. Bardenheirer BH, McNeill MM, Wodi AP, McNicholl JM, DeStefano F. Risk of Nontargeted Infectious Disease Hospitilisations Among US Children Following Inactivated and Live Vaccines, 2005-2014. Clin Infect Dis 2017; 65(5): 729-37.

52. O'Neill, L.A.J. and M.G. Netea, *BCG-induced trained immunity: can it offer protection against COVID-19?* Nat Rev Immunol, 2020. **20**(6): p. 335-337.

For peer review only

PRISMA 2009 Checklist

Section/topic	#	Checklist item	Reported on page #
**TITLE**			
Title	1	Identify the report as a systematic review, meta-analysis, or both.	1
**ABSTRACT**			
Structured summary	2	Provide a structured summary including, as applicable: background; objectives; data sources; study eligibility criteria, participants, and interventions; study appraisal and synthesis methods; results; limitations; conclusions and implications of key findings; systematic review registration number.	2-3
**INTRODUCTION**			
Rationale	3	Describe the rationale for the review in the context of what is already known.	4-7
Objectives	4	Provide an explicit statement of questions being addressed with reference to participants, interventions, comparisons, outcomes, and study design (PICOS).	7
**METHODS**			
Protocol and registration	5	Indicate if a review protocol exists, if and where it can be accessed (e.g., Web address), and, if available, provide registration information including registration number.	8
Eligibility criteria	6	Specify study characteristics (e.g., PICOS, length of follow-up) and report characteristics (e.g., years considered, language, publication status) used as criteria for eligibility, giving rationale.	8
Information sources	7	Describe all information sources (e.g., databases with dates of coverage, contact with study authors to identify additional studies) in the search and date last searched.	9
Search	8	Present full electronic search strategy for at least one database, including any limits used, such that it could be repeated.	9
Study selection	9	State the process for selecting studies (i.e., screening, eligibility, included in systematic review, and, if applicable, included in the meta-analysis).	9-10
Data collection process	10	Describe method of data extraction from reports (e.g., piloted forms, independently, in duplicate) and any processes for obtaining and confirming data from investigators.	10
Data items	11	List and define all variables for which data were sought (e.g., PICOS, funding sources) and any assumptions and simplifications made.	NA
Risk of bias in individual studies	12	Describe methods used for assessing risk of bias of individual studies (including specification of whether this was done at the study or outcome level), and how this information is to be used in any data synthesis.	10
Summary measures	13	State the principal summary measures (e.g., risk ratio, difference in means).	11
Synthesis of results	14	Describe the methods of handling data and combining results of studies, if done, including measures of consistency (e.g., I^2) for each meta-analysis.	11

For peer review only - http://bmjopen.bmj.com/site/about/guidelines.xhtml

PRISMA 2009 Checklist

Section/topic	#	Checklist item	Reported on page #
Risk of bias across studies	15	Specify any assessment of risk of bias that may affect the cumulative evidence (e.g., publication bias, selective reporting within studies).	11
Additional analyses	16	Describe methods of additional analyses (e.g., sensitivity or subgroup analyses, meta-regression), if done, indicating which were pre-specified.	NA
**RESULTS**			
Study selection	17	Give numbers of studies screened, assessed for eligibility, and included in the review, with reasons for exclusions at each stage, ideally with a flow diagram.	12
Study characteristics	18	For each study, present characteristics for which data were extracted (e.g., study size, PICOS, follow-up period) and provide the citations.	16-18
Risk of bias within studies	19	Present data on risk of bias of each study and, if available, any outcome level assessment (see item 12).	17
Results of individual studies	20	For all outcomes considered (benefits or harms), present, for each study: (a) simple summary data for each intervention group (b) effect estimates and confidence intervals, ideally with a forest plot.	14-17
Synthesis of results	21	Present results of each meta-analysis done, including confidence intervals and measures of consistency.	17
Risk of bias across studies	22	Present results of any assessment of risk of bias across studies (see Item 15).	17
Additional analysis	23	Give results of additional analyses, if done (e.g., sensitivity or subgroup analyses, meta-regression [see Item 16]).	NA
**DISCUSSION**			
Summary of evidence	24	Summarize the main findings including the strength of evidence for each main outcome; consider their relevance to key groups (e.g., healthcare providers, users, and policy makers).	19-20
Limitations	25	Discuss limitations at study and outcome level (e.g., risk of bias), and at review-level (e.g., incomplete retrieval of identified research, reporting bias).	21
Conclusions	26	Provide a general interpretation of the results in the context of other evidence, and implications for future research.	22
**FUNDING**			
Funding	27	Describe sources of funding for the systematic review and other support (e.g., supply of data); role of funders for the the systematic review.	23

From: Moher D, Liberati A, Tetzlaff J, Altman DG, The PRISMA Group (2009). Preferred Reporting Items for Systematic Reviews and Meta-Analyses: The PRISMA Statement. PLoS Med 6(7): e1000097. doi:10.1371/journal.pmed1000097

For more information, visit: **www.prisma-statement.org**.

For peer review only - http://bmjopen.bmj.com/site/about/guidelines.xhtml

BMJ Open

A Systematic Review of Non-Specific Effects of Pneumococcal and Haemophilus Vaccines in Children Aged Five Years and Under.

Journal:	*BMJ Open*
Manuscript ID	bmjopen-2023-077717.R1
Article Type:	Original research
Date Submitted by the Author:	24-Oct-2023
Complete List of Authors:	Geraghty, Keith; University of Limerick, School of Medicine<br>Rooney, Darragh; University of Limerick, School of Medicine<br>Watson, Chris; Queen's University Belfast Wellcome-Wolfson Institute for Experimental Medicine<br>Ledwidge, Mark; University College Dublin College of Health Sciences, gHealth Research Institute<br>Glynn, Liam; University of Limerick, School of Medicine<br>Gallagher, Joe; Irish College of General Practitioners, Global Health
**Primary Subject Heading**:	Global health
Secondary Subject Heading:	Health policy
Keywords:	Public health < INFECTIOUS DISEASES, Community child health < PAEDIATRICS, Paediatric infectious disease & immunisation < PAEDIATRICS, VIROLOGY

SCHOLARONE™
Manuscripts

I, the Submitting Author has the right to grant and does grant on behalf of all authors of the Work (as defined in the below author licence), an exclusive licence and/or a non-exclusive licence for contributions from authors who are: i) UK Crown employees; ii) where BMJ has agreed a CC-BY licence shall apply, and/or iii) in accordance with the terms applicable for US Federal Government officers or employees acting as part of their official duties; on a worldwide, perpetual, irrevocable, royalty-free basis to BMJ Publishing Group Ltd ("BMJ") its licensees and where the relevant Journal is co-owned by BMJ to the co-owners of the Journal, to publish the Work in this journal and any other BMJ products and to exploit all rights, as set out in our licence.

The Submitting Author accepts and understands that any supply made under these terms is made by BMJ to the Submitting Author unless you are acting as an employee on behalf of your employer or a postgraduate student of an affiliated institution which is paying any applicable article publishing charge ("APC") for Open Access articles. Where the Submitting Author wishes to make the Work available on an Open Access basis (and intends to pay the relevant APC), the terms of reuse of such Open Access shall be governed by a Creative Commons licence – details of these licences and which Creative Commons licence will apply to this Work are set out in our licence referred to above.

Other than as permitted in any relevant BMJ Author's Self Archiving Policies, I confirm this Work has not been accepted for publication elsewhere, is not being considered for publication elsewhere and does not duplicate material already published. I confirm all authors consent to publication of this Work and authorise the granting of this licence.

For peer review only

A Systematic Review of Non-Specific Effects of Pneumococcal and Haemophilus Vaccines in Children Aged Five Years and Under.

Authors

K Geraghty (School of Medicine UL);

Corresponding Author

School of Medicine,

University of Limerick, Limerick, Ireland

gerokeith@gmail.com

D Rooney (School of Medicine UL);

C Watson (School of Medicine UCD);

M Ledwidge (School of Medicine UCD);

L Glynn (School of Medicine UL);

J Gallagher (School of Medicine UCD)

For peer review only

ABSTRACT

Objective To determine the evidence for non-specific effects of the pneumococcal and haemophilus influenza vaccine in children aged five years and under.

Data sources A key word literature search of MEDLINE, Embase, The Cochrane Central Register of Controlled Trials, the European Union Clinical Trials Register, and clinicaltrials.gov up to June 2023.

Study eligibility criteria Randomised controlled trials, quasi-RCT or cohort studies.

Participants Children aged five or under.

Study appraisal and synthesis methods Studies were independently screened by two reviewers, with a third where disagreement arose. Risk of bias assessment was performed by one reviewer and confirmed by a second. Results were tabulated and a narrative description performed.

Results Four articles were identified and included in this review. We found a reduction in hospitalisations from influenza A (44%), Pulmonary TB (42%), metapneumovirus (45%), PIV Type 1-3 (44%), along with reductions in mortality associated with pneumococcal vaccine. No data on the haemophilus vaccine was found.

For peer review only

Conclusions and implications In this systematic review, we demonstrate that there is a reduction in particular viral infections in children aged five years and under who received the PCV9 vaccine which differ from those for which the vaccine was designed to protect against.

Review Registration The systematic review protocol was registered with the PROSPERO database (CRD42020146640)

For peer review only

For peer review only - http://bmjopen.bmj.com/site/about/guidelines.xhtml

STRENGTHS AND LIMITATIONS OF THIS STUDY

1. This systematic review explored the potential non-specific effects of two commonly administered vaccines in children aged five and under.
2. This systematic review was based on the systematic literature search following Preferred Reporting Items for Systematic review and Meta-Analysis.
3. Three of the articles reviewed were a post-hoc analysis of a phase III randomised, double-blind placebo-controlled PCV9 trial, using the same dataset.
4. No studies were found which explored the haemophilus vaccine in isolation.

For peer review only

INTRODUCTION

The beneficial effects of vaccination in reducing morbidity and mortality from the illnesses they are designed to prevent is widely reported with estimates that vaccines prevent approximately 6 million deaths worldwide annually [1,2]. Vaccination has attempted to reduce morbidity and mortality from major diseases such as diphtheria, tetanus, yellow fever, pertussis, haemophilus influenza type b disease, measles, mumps, rubella, typhoid and rabies, with the successful eradication of smallpox and near-complete eradication of poliomyelitis [3].

Pneumonia remains a major cause of morbidity and mortality worldwide. The World Health Organisation (WHO) estimates there to be 156 million cases of pneumonia each year in children younger than five years, with as many as 20 million requiring hospital admission, accounting for an estimated 0.935 million deaths every year [4, 5]. However, the number of pneumonia deaths in young children has almost halved between 2000 and 2015 [6]. This is likely attributable to improved provision of primary care through the Integrated Management of Childhood Illness programme, and increasing use of universal vaccination, including haemophilus influenza type B (Hib) and pneumococcal vaccines [7].

Vaccination with the Hib and pneumococcal conjugate vaccines protect children from invasive disease caused by these organisms. Routine vaccination has been shown to dramatically decrease the incidence of invasive Hib disease in children leading to a reduction in the number of hospital admissions [8, 9]. A study by Cowgill *et al*. [9] reported a reduction of the incidence of invasive Hib disease to 12% of its baseline level three years after the vaccine was

For peer review only - http://bmjopen.bmj.com/site/about/guidelines.xhtml

introduced. The 7-valent pneumococcal conjugate vaccine (PCV7) has been shown to reduce incidence rates of invasive pneumococcal disease by as much as 75% [10]. The introduction of the 13-valent pneumococcal conjugate vaccine (PCV13), replacing PCV7 in 2010, is likely to further reduce the incidence of pneumonia due to enhanced coverage of serotypes responsible for the majority of pneumococcal pneumonia cases in children worldwide [11]. GAVI, the Vaccine Alliance – previously the Global Alliance for Vaccines and Immunization – has been pivotal in the introduction of vaccination protocols enabling an estimated reduction of between 6 to 7.5 million cases of pneumonia and the avoidance of between 230,000 to 290,000 deaths since its introduction of its pneumococcal vaccine programme in 2007 [12].

Furthermore, there is a body of evidence that certain vaccines yield beneficial effects not just limited to the illnesses they are designed to prevent. These effects are commonly termed "non-specific" effects. Vaccination with measles-containing vaccines, Bacille Calmette-Guérin (BCG), and oral polio vaccine (OPV) have all been shown to reduce both hospitalisation rates and mortality in children with further research required to better understand the impact on mortality [13-15]. Non-specific beneficial effects of vaccination in pneumonia may be due to bacteria and viruses acting as co-pathogens in the aetiology of pneumonia [16]. Evidence also indicates that respiratory viruses contribute to bacterial infections, often leading to bacterial superinfections [16]. Non-specific beneficial effects of vaccination may be due to the results of altered immune system memory, leading to a reduction in all-cause mortality [17, 18]. One study proposes that live vaccines can induce innate immune training, producing pro-inflammatory responses to unrelated antigens [19]. However, there is contradictory evidence, with one systematic review demonstrating that receipt of the diphtheria pertussis tetanus (DPT) vaccine was shown to be associated with an increase in all-cause mortality. This should

For peer review only - http://bmjopen.bmj.com/site/about/guidelines.xhtml

be interpreted with caution as all ten studies within the analysis were observational and classed as having a “high risk of bias.” [19, 20].

In recent years, researchers have offered a compelling argument for a radical change in the current vaccine paradigm [21], and newly suggested principles could mean a drastic overhaul in how countries administer their vaccines programmes. For example, it is suggested that live vaccines yield non-specific effects which are beneficial [22-26], while non-live vaccines may have detrimental non-specific effects, especially for females [27, 28] In addition, it is proposed that non-specific effects of vaccines are determined by their most recent vaccine [29].

Further work is required to determine the presence or absence of non-specific effects of newer vaccines such as pneumococcus and haemophilus influenza which were more recently added to schedules internationally. In this study, we aim to determine if the administration of pneumococcal and/or Hib vaccines in infancy is associated with an effect on survival or hospitalisations from infections other than those conditions that the vaccine is designed to prevent in children up to five years of age. Furthermore, we aim to explore if the administration of pneumococcal and/or Hib vaccines in infancy are associated with an effect on severity of the illness as defined by the authors, or hospitalisations for infections other than those conditions that the vaccine is designed to prevent in children up to five years of age.

For peer review only - http://bmjopen.bmj.com/site/about/guidelines.xhtml

METHOLODOLOGY

The systematic review protocol was registered with the PROSPERO database (CRD42020146640), which guided the authors in conducting this review. The preferred reporting items for systematic reviews and meta-analyses (PRISMA) statement [30] was used to guide the reporting and conduct of the review.

Eligibility Criteria

To be eligible for inclusion, studies had to meet certain predetermined criteria outlined in the review protocol. The population of interest was children aged five and under. Haemophilus vaccines along with any formulation of the pneumococcal vaccine, including PCV7, PCV13, and PCV23 were included. Studies reporting survival, all-cause mortality, or deaths from infections other than those conditions that the vaccine was designed to prevent, or studies reporting deaths from all causes (e.g., all-cause mortality, child survival) or severity as defined by the authors (e.g., hospitalisation) were eligible for inclusion. Only randomised controlled trials (RCTs), quasi-RCTs or cohort studies written in English were included in this review.

Exclusion Criteria

Ecological studies, uncontrolled studies (i.e., case reports and case series studies), “case-only” studies, and self-controlled case series studies were excluded from this review, as these studies provide less reliable data for assessing non-specific effects of vaccine on hospitalisations and mortality. Additionally, animal and laboratory studies were excluded. Studies which included adults or children over the age of five, or those which did not report children under the age of five separately, were not included in this review.

For peer review only

Data Management

Results from the literature search were directly imported into the online Rayyan software package [31]. This was used for screening through the citations and abstracts from the electronic databases, as well as the full-text articles indicated via the search. Two reviewers independently screened the literature search results for inclusion. They then independently reviewed the full text of potentially relevant articles and screened them to determine inclusion using the same inclusion and exclusion criteria. In cases of disagreement a third reviewer acted as arbitrator.

For peer review only

Search Strategy

The search strategy was jointly developed and tested during the protocol phase and was subsequently finalised by reviewers prior to commencing the search. Medical subject headings and text words related to children, pneumococcal and haemophilus influenza vaccination were used. Electronic databases MEDLINE (OVID interface, 1948 onwards)[32], EMBASE (OVID interface, 1980 onwards)[33] and Pubmed were each searched using the predetermined search terms. The Cochrane Central Register of Controlled Trials (Wiley interface, current issue)[34] and European Union Clinical Trials Register (EURACT) were searched to supplement the search. These databases were last searched end of June 2023. All reference lists of included studies were scrutinised and reviewed to further identify relevant reviews not found during the database search. Finally, a bibliography of included articles was circulated to experts in the field to ensure literature saturation. The flow chart of the study identification process is illustrated in figure 1.

For peer review only - http://bmjopen.bmj.com/site/about/guidelines.xhtml

Data Collection Process

To ensure data accuracy, two reviewers independently abstracted the data using a standardised data abstraction form. This form was developed during the protocol phase and was trialled and refined prior to commencement of the review.

Risk of Bias Assessment

Two researchers independently assessed risk of bias using the Cochrane Collaboration risk of bias tool (Cochrane RoB2 version)[35]. Guided by the recommendations of the Cochrane handbook, seven methodological items were used to assess each individual study's risk of bias, including randomised sequence generation, elective reporting, incomplete outcome data, blinding of outcome assessment, blinding of participants and personnel, method of concealing allocation, and other potential biases. Risk of bias was subsequently classified as 'low risk, 'unclear,' or 'high risk.' Discussion and consultation with a third reviewer took place in the case of disagreement between the two primary assessors.

Data Synthesis

To effectively synthesise the data obtained, data was grouped together and tabulated. Textual descriptions were provided. Studies were contrasted, and a narrative on reported outcomes, effectiveness of studies, and design strengths and weaknesses were presented.

Patient and Public Involvement

Patients or the public were not involved in the design, or conduct, or reporting, or dissemination plans of our research.

For peer review only - http://bmjopen.bmj.com/site/about/guidelines.xhtml

Ethical Approval

This review does not require ethical approval as authors have retrieved and synthesised previously published literature.

For peer review only

RESULTS

We retrieved 1,341 articles from MEDLINE, EMBASE, and PubMed electronic databases using our search strategy. Rayyan software automatically removed duplicate records, and suggested articles which were similar in title or author, allowing manual removal of any duplicates not successfully detected by the software package. We had prior knowledge of one article which met our inclusion criteria, which was therefore included in the screening process. One further article was suggested by an expert in the field, and this was also included. After duplicates were removed, 727 records remained and were subsequently screened by two reviewers independently. Of these, 717 were excluded based on screening abstracts and titles. Ten full-text articles were assessed for eligibility, using the eligibility form. Six of these were excluded as they did not meet inclusion criteria *(n = 3*), or they did not evaluate the described intervention *(n = 3).* Scanning references from the remaining articles did not reveal any new articles. Therefore, four studies were included in our systematic review. Three studies were post-hoc analyses of the same randomised control trial dataset. One study was a randomised control study.

All four studies were associated with the use of the 9-valent pneumococcal conjugate vaccine. No study involving the use of the haemophilus influenza vaccine was identified.

Considering the use of common datasets, 40,100 children under the age of five were involved in these studies. Summaries of the main findings of each study are outlined in *Table 1.* Results of these studies are summarised in *Table 2.*

For peer review only

Table 1: Study Characteristics

Authors	Study Type	Country	Population	Subpopulation	Vaccine	Control	Other Vaccines concurrently administered
Moore DP, Klugman KP, Madhi SA (2010)[36]	Post-hoc analysis of a phase III randomised, double-blind placebo-controlled PCV9 trial	South Africa	39,836 children, aged 84 days and under	HIV-infected and HIV-uninfected	9-valent pneu- mococcal vaccine or a placebo<br><br>[2 µg of capsular polysac-charide (serotypes 1, 4, 5, 9V, 14, 19F, and 23F), 4 µg of serotype 6B, and 2 µg of oligosaccharide 18C]	Haemophilus influenzae type b conjugate vaccine	Diphtheria, tetanus, and whole-cell pertussis (DTwP, Aventis Pasteur); hepatitis B (Hepaccine-B, Cheil Sugar Organization); and oral live, trivalent poliovirus types 1, 2, and 3 (Poli- oral, Biovac).
Madhi SA, Klugman KP, & The Vaccine Trialist Group (2004)[37]	Post-hoc analysis of a phase III randomised, double-blind placebo-controlled PCV9 trial	South Africa	39,836 children, aged 84 days and under	HIV-infected and HIV-uninfected	9-valent pneu- mococcal vaccine or a placebo<br><br>[2 µg of capsular polysac-charide (serotypes 1, 4, 5, 9V, 14, 19F, and 23F), 4 µg of serotype 6B, and 2 µg of oligosaccharide 18C]	Haemophilus influenzae type b conjugate vaccine	Diphtheria, tetanus, and whole-cell pertussis (DTwP, Aventis Pasteur); hepatitis B (Hepaccine-B, Cheil Sugar Organization); and oral live, trivalent poliovirus types 1, 2, and 3 (Poli- oral, Biovac).
Madhi, S.A., et al. (2006)[38]	Post-hoc analysis of a phase III randomised, double-blind placebo-controlled trial	South Africa	39,836 children (aged 60-84 days)	HIV-infected and HIV-uninfected	9-valent pneu- mococcal vaccine or a placebo<br><br>[2 µg of capsular polysac-charide (serotypes 1, 4, 5, 9V, 14, 19F, and 23F), 4 µg of serotype 6B, and 2 µg of oligosaccharide 18C]	Haemophilus influenzae type b conjugate vaccine	Diphtheria, tetanus, and whole-cell pertussis (DTwP, Aventis Pasteur); hepatitis B (Hepaccine-B, Cheil Sugar Organization); and oral live, trivalent poliovirus types 1, 2, and 3 (Poli- oral, Biovac).
Dagan R, Sikuler-Cohen M, Zamir O, Janco J, Givon-Lavi N, Fraser D (2001)[39]	Randomised, double-blind placebo-controlled PCV9 trial	Israel	264 children (aged 12-35 months)	-	9-valent pneumococcal conjugate vaccine<br><br>[2 µg of capsular polysac-charide (serotypes 1, 4, 5, 9V, 14, 18C, 19F and 23F), 4 µg of serotype 6B, coupled to diphtheria toxin CRM197]	Meningococcus group C conjugate vaccine	Not reported

Study	Subpopulation	Illness	Outcome Measured	Efficacy (95% CI)	*P*
Moore DP, Klugman KP, Madhi SA (2010)[36]	HIV-uninfected	First Episode Culture-Confirmed PTB	All-Cause Hospitalisations	35.3 (-38.1-69.7)	0.2562
		All categories of first episode PTB		0.0 (-40.9-29.1)	0.9983
		All culture-confirmed PTB		38.9 (-29.3-71.1)	0.1931
	HIV-infected	First Episode Culture-Confirmed PTB		47.3 (8.6-69.6)	0.0203
		All categories of first episode PTB		13.2 (-10.4-31.8)	0.2475
		All culture-confirmed PTB		43.6 (5.5-66.4)	0.0274
	Overall	First Episode Culture-Confirmed PTB		43.4 (11.5-63.8)	0.0114
		All categories of first episode PTB		8.8 (-11.7-25.5)	0.9983
		All culture-confirmed PTB		42.1 (11.2-62.3)	0.0113
Madhi SA, Klugman KP, & The Vaccine Trialist Group (2004)[37]	HIV-uninfected	Any Virus-Associated Pneumonia	All-Cause Hospitalisations	33 (15-48)	0.0008
		Influenza A		34 (-14-62)	0.1
		RSV		32 (6-50)	0.02
		PIV Types 1-3		41 (-10-68)	0.09
		Adenovirus		31 (-62-70)	0.4
	HIV-infected	Any Virus-Associated Pneumonia		0.2 (-14-47)	23
		Influenza A		57 (7-80)	0.03
		RSV		-30 (-140-31)	0.4
		PIV Types 1-3		50 (-17-78)	0.1
		Adenovirus		-150 (-1188-51)	0.3
	Overall	Any Virus-Associated Pneumonia		31 (15-43)	0.0004
		Influenza A		45 (14-64)	0.01
		RSV		22 (-3-41)	0.08
		PIV Types 1-3		44 (8-66)	0.02
		Adenovirus		7 (-94-55)	0.9
Madhi, S.A., et al. (2006)[38]	HIV-uninfected	**Human Metapneumovirus-Associated LRTI**	All-Cause Hospitalisations		
		<6.0 months		6 (-83-51)	0.87
		6.1-12 months		52 (7-75)	0.027
		12.1-24 months		33 (-17-62)	0.16
		>24 months		47 (-13-76)	0.09
	HIV-infected	<6.0 months		34 (-295-89)	0.65

For peer review only

For peer review only - http://bmjopen.bmj.com/site/about/guidelines.xhtml

		6.1-12 months<br>12.1-24 months<br>>24 months		43 (-93-83)<br>58 (-64-89)<br>42 (-46-77)	0.36<br>0.20<br>0.24
	Overall	<6.0 months<br>6.1-12 months<br>12.1-24 months<br>>24 months		14 (-59-53)<br>52 (14-73)<br>40 (-1-64)<br>45 (1-70)	0.63<br>0.012<br>0.053<br>0.04
Dagan R, Sikuler-Cohen M, Zamir O, Janco J, Givon-Lavi N, Fraser D (2001)[39]	-	**URI**<br><36 months<br>>36 months<br>Overall<br><br>**All-cause LRTI**<br><36 months<br>>36 months<br>Overall<br><br>**Otitis Media**<br><36 months<br>>36 months<br>Overall	Hospitalisations	 17 (0.7-0.99)<br>14 (0.73-1.02)<br>15 (0.76-0.96)<br><br><br>23 (0.62-0.95)<br>9 (0.73-1.12)<br>16 (0.72-0.98)<br><br><br>23 (0.58-1.03)<br>12 (0.65-1.20)<br>17 (0.67-1.02)	 0.036<br>0.082<br>0.009<br><br><br>0.015<br>0.371<br>0.024<br><br><br>0.075<br>0.425<br>0.078

Table 2: Results

For peer review only - http://bmjopen.bmj.com/site/about/guidelines.xhtml

Three of the studies [36-38], all of which undertook a *post-hoc* analysis of a prospective randomised, double-blind placebo-controlled PCV9 efficacy study demonstrated a reduction in hospitalisations from infections other than those that the pneumococcal conjugate vaccine was designed to prevent against. The trial, conducted in South Africa, enrolled 39,836 children between 1 March 1998 and October 2000. Participants received either PCV9 or a control, and investigators and laboratory staff remained blinded to the randomisation throughout the surveillance phase. The mortality rate was reduced by 5 percent among all children (*p=0.58),* and by 6 percent among HIV-infected children *(p=0.63)* in this study.

Upon analysis of this dataset, Moore *et al.*[36] determined that there was a reduction in hospitalisation for culture-confirmed pulmonary tuberculosis (PTB) of 43.4% in the vaccine study group versus the control group (95% CI: 11.5-63.8; *p=0.0114.)* In HIV-infected children, the risk of being hospitalised with a lower respiratory tract infection (LRTI) was 47.3% lower (95% CI: 8.6-69.6; *p=0.0203*) in PCV9 recipients compared to the placebo recipients. Although not statistically significant, a similar trend was observed in children uninfected by HIV (relative risk reduction 35.3% [95% CI: -38.1-69.7]; *p*=0.2562). Where available, intention-to-treat (ITT) results are described as it reflects the practical clinical scenario and gives an unbiased estimate of treatment effect[40].

In their analysis of the same dataset, Madhi *et al.*[37] show that in all children (i.e. those with or without HIV infection), PCV9 reduced pneumonias associated with any of the identified viruses by 31% (95% CI: 15-43; *p=0.0004*). Furthermore, there were similar point estimates of efficacy with Influenza A (45%; 95 CI: 14-64; *p=0.01)*, parainfluenza virus type 1-3 (44%;

For peer review only - http://bmjopen.bmj.com/site/about/guidelines.xhtml

95% CI: 3-64%; *p=0.01)* and respiratory syncytial virus (22%; 95 CI: -3-41; *p=0.08*) in all children.

In a further study, Madhi *et al.* [38] demonstrated a reduction in incidence of human metapneumovirus in both HIV-infected and HIV-unaffected children who received the PCV9 vaccine, (reduction of 53% [95% CI: 3-77; *p=0.035*] vs 45% reduction [95% CI: 19-62; *p=0.002*], in the per protocol analysis, respectively). In this study, the authors also note a significant reduction in the incidence of clinical pneumonia among vaccine recipients overall (58; 95% CI 34-73; *p=0.0002*).

Dagan *et al*.[39] conducted a small randomised, double-blind, placebo-controlled study in Israel, whereby 264 toddlers received either PCV9, or a control (meningococcal conjugate vaccine). This study revealed a 23% reduction (95% CI: 0.62-0.95; *p=0.015*) in all-cause lower respiratory infection in children under 36 months compared with placebo recipients. A 17% reduction in upper respiratory tract infection was observed in the same age group (95% CI: 0.7-0.99; *p=0.036*). Although there was a similar trend in children >36 months, this was not statistically significant.

We used the Cochrane tool for assessing risk of bias in clinical trials (RoB2) [35] for randomised studies. All studies were judged based on predetermined criteria, as discussed in the Methods sections of this paper. The studies included in this review were deemed low risk of bias. Furthermore, we considered any potential confounders and based on the location of the trials, found the socioeconomic status of the population to be a potential confounder for both the South African and Israeli studies. Certain populations are more likely to live in

For peer review only

For peer review only - http://bmjopen.bmj.com/site/about/guidelines.xhtml

overcrowded communities, potentially subjecting them to a higher burden of disease [41]. Furthermore, we felt socioeconomic status was important as it may determine inequalities in healthcare access [41]. In all papers, there was no discussion which raised possible implications for vaccinating children in areas where disease may be more prevalent.

For peer review only

For peer review only - http://bmjopen.bmj.com/site/about/guidelines.xhtml

DISCUSSION

Reports to date have determined the efficacy of PCV9 in the reduction of nasopharyngeal pneumococcal carriage [42], invasive infection [43] and mucosal infections [43]. In this systematic review, we demonstrate that in some cohorts, there is a reduction in particular viral infections in children under the age of five who received the PCV9 vaccine, which differ from those for which the vaccine was designed to protect against.

Reduction in the incidence of hospitalisation from other diseases was reported most frequently. *Post-hoc* analyses from a phase III randomised control trial in South Africa reveal a reduction in culture-confirmed pulmonary TB [36], influenza A [37], parainfluenza type 1-3 [37], and human metapneumovirus [38] in all children, irrespective of their HIV status. In a smaller RCT in Israel [39], a reduction in all-cause lower respiratory tract infections was determined in day-care centre attendees.

The authors of the phase III randomised control trial included analysis of HIV-infected versus non-infected children, which allowed post-hoc analysis of these subgroups. While the hospital reductions mentioned above include all children, the reduction of PTB in vaccinee recipients is only statistically significant in HIV-infected children, and overall [36]. Furthermore, the reduction of RSV was only significant in all-children, and those not infected with HIV [37]. All-cause mortality did not differ between groups in this trial.

For peer review only - http://bmjopen.bmj.com/site/about/guidelines.xhtml

While included trials have a relatively equal representation of both sexes in their population, there is no analysis of potential non-specific effects of the respective vaccines on males versus females. As described, several non-live vaccines, namely DPT [44], inactive polio vaccine [45], hepatitis B vaccine [46], measles and yellow fever vaccine [47] are associated with increased female mortality. Given the pneumococcal vaccine is inactivated, further work exploring the sex-differential is warranted.

Interestingly, the control used in the randomised control trial in South Africa was the Haemophilus influenzae type b conjugate vaccine, the second vaccine we sought to explore in this review. This was given so those children in the control group could infer some benefit. It is therefore likely that these subsets would receive some protection against haemophilus influenza. No study was identified which sought to explore non-specific effects in the Hib vaccine in isolation, and further research is required in this domain.

The South African trial reported on other vaccinations children received as part of their routine schedule, although did not outline the sequence in which these vaccines were administered. Unfortunately, this information was omitted from the Dagan study. Certain combinations of vaccines are associated with increased mortality (e.g., DTP and measles vaccine) [48], whereas others are associated with lower mortality (e.g. DTP and OPV) [49]. In addition, there is evidence that combining non-live and live vaccines can have a detrimental effect on mortality [50]. Therefore, further detail regarding the sequence of vaccines is crucial, as it may impact efficacy and mortality.

For peer review only - http://bmjopen.bmj.com/site/about/guidelines.xhtml

This study has several limitations. Most notably, three of the articles reviewed were a *post-hoc* analysis of the same dataset, which used the same population. Post-hoc analyses typically describes findings for which the parent study was not designed to explore. In addition, the high proportion of studies in this area which come from common data sets is a weakness of this review. As discussed, analysis to include sex-differential and sequencing of vaccines is missing from these studies.

The findings of this review do however add to the body of evidence that certain vaccines infer additional non-specific effects. The most widely reported of these vaccines are the BCG and oral polio vaccine [14, 15], with some currently hypothesising that the BCG vaccine may in fact offer protection against the SARS-CoV-2 virus [51].

Vaccination programmes have demonstrated wide-ranging benefits, however some of these are poorly defined. For public health officials and those deciding on health policy, there is a need to prioritise vaccines which have an identifiable and preventable disease burden. Consideration of those non-specific effects which may impact upon morbidity and mortality are important in deciding upon novel vaccination strategies.

For peer review only

CONCLUSION

This review highlights the paucity of literature exploring the non-specific effects of the pneumococcal conjugate vaccine and haemophilus vaccine. While limited studies have demonstrated a reduction in infections other than those which the vaccine was designed to protect against, substantial clinical trials are required to solidify these findings.

For peer review only

For peer review only - http://bmjopen.bmj.com/site/about/guidelines.xhtml

Funding

This study was conducted with the financial support of the Bill and Melinda Gates Foundation (OPP113957) and Science Foundation Ireland (SFI) and the Department of Foreign Affairs (DFA) under the SDG Challenge Grant Number SFI/21/FIP/SDG/9948

Author Contributorship

Geraghty, K – acquisition of data; analysis and interpretation of data; drafting of manuscript, primary reviewer

Rooney, D – conception and design of study; protocol design and registration; third reviewer

Watson, C – reviewer of manuscript

Ledwidge, Mark T – reviewer of manuscript

Glynn, L – reviewer of manuscript

Gallagher, J – conception and design of study; protocol design and registration; critical revisions of manuscript; primary reviewer

Conflict of Interest

All authors declare that they have no conflicts of interest.

Data Sharing Statement

Data is available upon reasonable request.

For peer review only

REFERENCES

1. Andre, F.E., et al., *Vaccination greatly reduces disease, disability, death and inequity worldwide.* Bull World Health Organ, 2008. **86**(2): p. 140-6.
2. Ehreth, J., *The global value of vaccination.* Vaccine, 2003. **21**(7-8): p. 596-600.
3. Plotkin, S., *History of vaccination.* Proc Natl Acad Sci U S A, 2014. **111**(34): p. 12283-7.
4. Rudan, I., et al., *Epidemiology and etiology of childhood pneumonia.* Bull World Health Organ, 2008. **86**(5): p. 408-16.
5. Liu, L., et al., *Global, regional, and national causes of child mortality in 2000-13, with projections to inform post-2015 priorities: an updated systematic analysis.* Lancet, 2015. **385**(9966): p. 430-40.
6. McAllister, D.A., et al., *Global, regional, and national estimates of pneumonia morbidity and mortality in children younger than 5 years between 2000 and 2015: a systematic analysis.* Lancet Glob Health, 2019. **7**(1): p. e47-e57.
7. Gove, S., *Integrated management of childhood illness by outpatient health workers: technical basis and overview. The WHO Working Group on Guidelines for Integrated Management of the Sick Child.* Bull World Health Organ, 1997. **75 Suppl 1**(Suppl 1): p. 7-24.
8. *Progress toward elimination of Haemophilus influenzae type b invasive disease among infants and children--United States, 1998-2000.* MMWR Morb Mortal Wkly Rep, 2002. **51**(11): p. 234-7.
9. Cowgill, K.D., et al., *Effectiveness of Haemophilus influenzae type b Conjugate vaccine introduction into routine childhood immunization in Kenya.* Jama, 2006. **296**(6): p. 671-8.

For peer review only - http://bmjopen.bmj.com/site/about/guidelines.xhtml

10. *Direct and indirect effects of routine vaccination of children with 7-valent pneumococcal conjugate vaccine on incidence of invasive pneumococcal disease--United States, 1998-2003.* MMWR Morb Mortal Wkly Rep, 2005. **54**(36): p. 893-7.

11. Resti, M., et al., *Community-acquired bacteremic pneumococcal pneumonia in children: diagnosis and serotyping by real-time polymerase chain reaction using blood samples.* Clin Infect Dis, 2010. **51**(9): p. 1042-9.

12. The Vaccine Alliance. *GAVI*. 2020 [cited 2020 06/09/2020]; Available from: https://www.gavi.org/.

13. Aaby, P., et al., *Non-specific effects of standard measles vaccine at 4.5 and 9 months of age on childhood mortality: randomised controlled trial.* Bmj, 2010. **341**: p. c6495.

14. de Castro, M.J., J. Pardo-Seco, and F. Martinón-Torres, *Nonspecific (Heterologous) Protection of Neonatal BCG Vaccination Against Hospitalization Due to Respiratory Infection and Sepsis.* Clin Infect Dis, 2015. **60**(11): p. 1611-9.

15. Lund, N., et al., *The Effect of Oral Polio Vaccine at Birth on Infant Mortality: A Randomized Trial.* Clin Infect Dis, 2015. **61**(10): p. 1504-11.

16. Peltola, V.T. and J.A. McCullers, *Respiratory viruses predisposing to bacterial infections: role of neuraminidase.* Pediatr Infect Dis J, 2004. **23**(1 Suppl): p. S87-97.

17. Saeed, S., et al., *Epigenetic programming of monocyte-to-macrophage differentiation and trained innate immunity.* Science, 2014. **345**(6204): p. 1251086.

18. Benn, C.S., et al., *A small jab - a big effect: nonspecific immunomodulation by vaccines.* Trends Immunol, 2013. **34**(9): p. 431-9.

19. Kleinnijenhuis J, Quintin J, Preijers F, et al. *Bacille Calmette-Guerin induces NOD2-dependent nonspecific protection from reinfection via epigenetic reprogramming of monocytes.* Proc Natl Acad Sci U S A 2012; 109(43): 17537-42.

For peer review only - http://bmjopen.bmj.com/site/about/guidelines.xhtml

20. Yung, C.F., *Non-specific effects of childhood vaccines.* Bmj, 2016. **355**: p. i5434.

21. Be Benn, C. S., Fisker, A. B., Rieckmann, A., Sørup, S., & Aaby, P. (2020). *Vaccinology: time to change the paradigm?.* The Lancet. Infectious diseases, 20(10), e274–e283nn, C. S., Fisker, A. B., Rieckmann, A., Sørup, S., & Aaby, P. (2020). Vaccinology: time to change the paradigm?. The Lancet. Infectious diseases, 20(10), e274–e283

22. Sorup S, Benn CS, Poulsen A, Krause TG, Aaby P, Ravn H. *Live vaccine against measles, mumps, and rubella and the risk of hospital admissions for nontargeted infections.* JAMA 2014; 311(8): 826-35.

23. La Torre G, Saulle R, Unim B, et al. *The effectiveness of measles-mumps-rubella (MMR) vaccination in the prevention of pediatric hospitalizations for targeted and untargeted infections: A retrospective cohort study*. Hum Vaccin Immunother 2017; 13(8): 1879-83.

24. Upfill-Brown A, Taniuchi M, Platts-Mills JA, et al. *Nonspecific Effects of Oral Polio Vaccine on Diarrheal Burden and Etiology Among Bangladeshi Infants*. Clin Infect Dis 2017; 65(3): 414-9.

25. Seppala E, Viskari H, Hoppu S, et al. *Viral interference induced by live attenuated virus vaccine (OPV) can prevent otitis media.* Vaccine 2011; 29(47): 8615-8.

26. Rieckmann A, Villumsen M, Sorup S, et al. *Vaccinations against smallpox and tuberculosis are associated with better long-term survival: a Danish case-cohort study 1971-2010*. Int J Epidemiol 2017; 46(2): 695-705.

27. Aaby P, Jensen H, Samb B, et al. *Differences in female-male mortality after high-titre measles vaccine and association with subsequent vaccination with diphtheria-tetanus-pertussis and inactivated poliovirus: reanalysis of West African studies.* Lancet 2003; 361(9376): 2183-8.

For peer review only - http://bmjopen.bmj.com/site/about/guidelines.xhtml

28. Holt EA, Moulton LH, Siberry GK, Halsey NA. *Differential mortality by measles vaccine titer and sex*. J Infect Dis 1993; 168(5): 1087-96

29. Aaby P, Ravn H, Benn CS, et al. Randomized Trials Comparing Inactivated Vaccine After Medium- or High-titer Measles Vaccine With Standard Titer Measles Vaccine After Inactivated Vaccine: A Meta-analysis. Pediatr Infect Dis J 2016; 35(11): 1232-41.

30. Moher, D., et al., *Preferred reporting items for systematic review and meta-analysis protocols (PRISMA-P) 2015 statement.* Syst Rev, 2015. **4**(1): p. 1.

31. Ouzzani, M., et al., *Rayyan-a web and mobile app for systematic reviews.* Syst Rev, 2016. **5**(1): p. 210.

32. MEDLINE. *MEDLINE*. 2020; Available from: https://www.ncbi.nlm.nih.gov/pubmed.

33. EMBASE. *EMBASE*. 2020; Available from: https://www.embase.com/login.

34. Cochrane, *Cochrane Central Register of Controlled Trials*. 2020.

35. Sterne, J.A.C., et al., *RoB 2: a revised tool for assessing risk of bias in randomised trials.* Bmj, 2019. **366**: p. l4898.

36. Moore, D.P., K.P. Klugman, and S.A. Madhi, *Role of Streptococcus pneumoniae in hospitalization for acute community-acquired pneumonia associated with culture-confirmed Mycobacterium tuberculosis in children: a pneumococcal conjugate vaccine probe study.* Pediatr Infect Dis J, 2010. **29**(12): p. 1099-04.

37. Madhi, S.A. and K.P. Klugman, *A role for Streptococcus pneumoniae in virus-associated pneumonia.* Nat Med, 2004. **10**(8): p. 811-3.

38. Madhi, S.A., et al., *Pneumococcal coinfection with human metapneumovirus.* J Infect Dis, 2006. **193**(9): p. 1236-43.

For peer review only - http://bmjopen.bmj.com/site/about/guidelines.xhtml

39. Dagan, R., et al., *Effect of a conjugate pneumococcal vaccine on the occurrence of respiratory infections and antibiotic use in day-care center attendees.* Pediatr Infect Dis J, 2001. **20**(10): p. 951-8.

40. Heritier, S.R., V.J. Gebski, and A.C. Keech, *Inclusion of patients in clinical trial analysis: the intention-to-treat principle.* Med J Aust, 2003. **179**(8): p. 438-40

41. Public Health England. Beyond the data: Understanding the impact of COVID-19 on BAME groups. 2020

42. Mbelle, N., et al., *Immunogenicity and impact on nasopharyngeal carriage of a nonavalent pneumococcal conjugate vaccine.* J Infect Dis, 1999. **180**(4): p. 1171-6.

43. Black, S., et al., *Efficacy, safety and immunogenicity of heptavalent pneumococcal conjugate vaccine in children. Northern California Kaiser Permanente Vaccine Study Center Group.* Pediatr Infect Dis J, 2000. **19**(3): p. 187-95.

44. Aaby P, Mogensen SW, Rodrigues A, Benn CS. Evidence of Increase in Mortality After the Introduction of Diphtheria-Tetanus-Pertussis Vaccine to Children Aged 6-35 Months in Guinea-Bissau: A Time for Reflection? *Front Public Health* 2018; **6**(79): 79.

45. Aaby P, Garly ML, Nielsen J, et al. Increased female-male mortality ratio associated with inactivated polio and diphtheria-tetanus-pertussis vaccines: Observations from vaccination trials in Guinea-Bissau. *Pediatr Infect Dis J* 2007; **26**(3): 247-52.

46. Garly ML, Jensen H, Martins CL, et al. Hepatitis B vaccination associated with higher female than male mortality in Guinea-bissau: an observational study. *Pediatr Inf Dis J* 2004; **23**(12): 1086-92.

47. Fisker AB, Biering-Sorensen S, Lund N, et al. Contrasting female-male mortality ratios after routine vaccinations with pentavalent vaccine versus measles and yellow fever vaccine. A cohort study from urban Guinea-Bissau. *Vaccine* 2016; **34**(38): 4551-7.

For peer review only - http://bmjopen.bmj.com/site/about/guidelines.xhtml

48. Higgins JP, Soares-Weiser K, Lopez-Lopez JA, et al. *Association of BCG, DTP, and measles containing vaccines with childhood mortality: a sytematic review*. BMJ 2016; 355:i5170.

49. Mogensen SW, Andersen A, Rodrigues A, Benn CS, Aaby P. The Introduction of Diphtheria-Tetanus-Pertussis and Oral Polio Vaccine Among Young Infants in Urban African Community: A Natural Experiment. EBioMedicine 2017; 17: 192-8.

50. Bardenheirer BH, McNeill MM, Wodi AP, McNicholl JM, DeStefano F. Risk of Nontargeted Infectious Disease Hospitilisations Among US Children Following Inactivated and Live Vaccines, 2005-2014. Clin Infect Dis 2017; 65(5): 729-37.

51. O'Neill, L.A.J. and M.G. Netea, *BCG-induced trained immunity: can it offer protection against COVID-19?* Nat Rev Immunol, 2020. **20**(6): p. 335-337.

For peer review only

Figure Legend

Figure 1

Preferred Reporting Items for Systematic Reviews and Meta-Analyses flow chart.

For peer review only

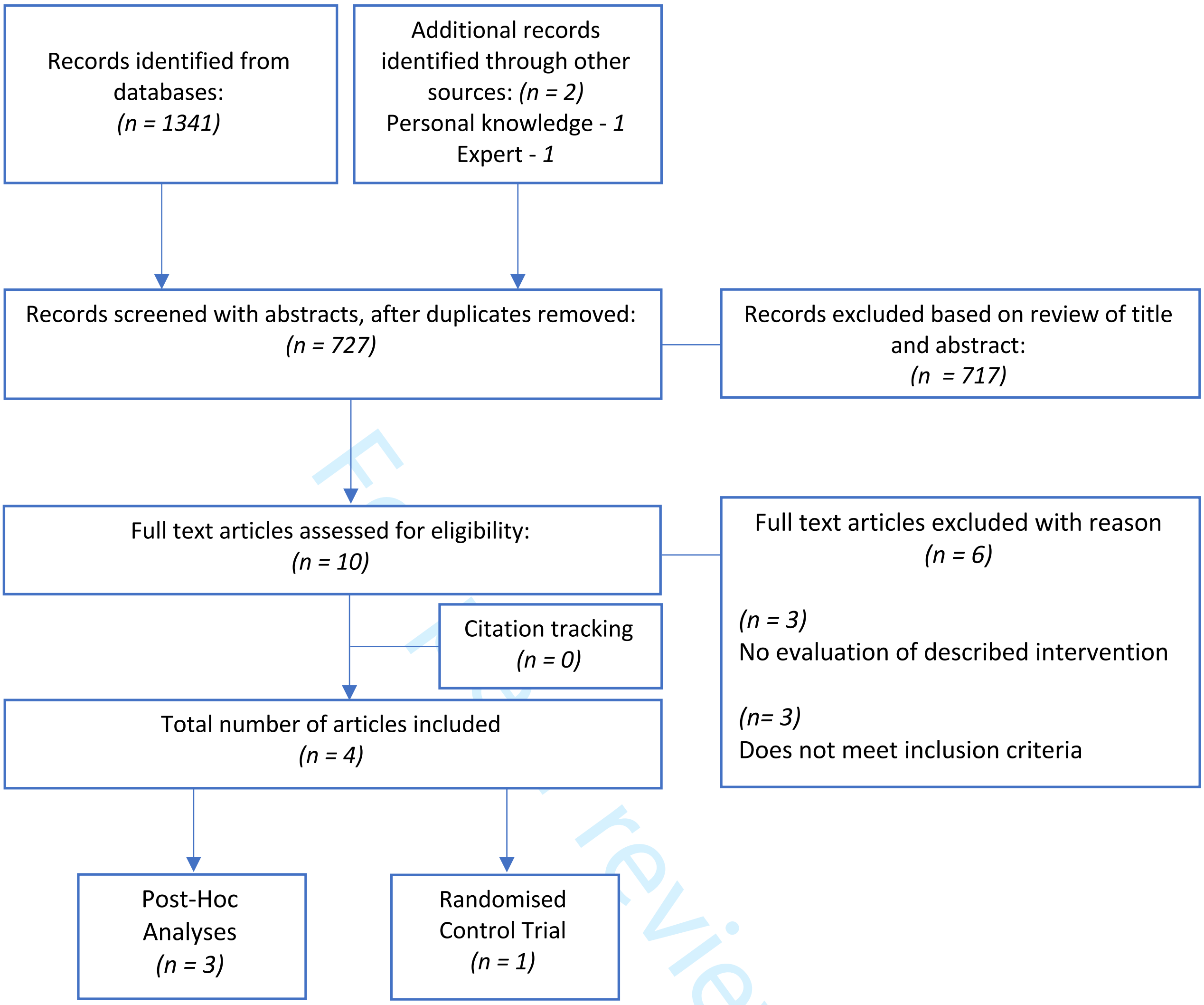

For peer review only

PRISMA 2009 Checklist

Section/topic	#	Checklist item	Reported on page #
**TITLE**			
Title	1	Identify the report as a systematic review, meta-analysis, or both.	1
**ABSTRACT**			
Structured summary	2	Provide a structured summary including, as applicable: background; objectives; data sources; study eligibility criteria, participants, and interventions; study appraisal and synthesis methods; results; limitations; conclusions and implications of key findings; systematic review registration number.	2-3
**INTRODUCTION**			
Rationale	3	Describe the rationale for the review in the context of what is already known.	4-7
Objectives	4	Provide an explicit statement of questions being addressed with reference to participants, interventions, comparisons, outcomes, and study design (PICOS).	7
**METHODS**			
Protocol and registration	5	Indicate if a review protocol exists, if and where it can be accessed (e.g., Web address), and, if available, provide registration information including registration number.	8
Eligibility criteria	6	Specify study characteristics (e.g., PICOS, length of follow-up) and report characteristics (e.g., years considered, language, publication status) used as criteria for eligibility, giving rationale.	8
Information sources	7	Describe all information sources (e.g., databases with dates of coverage, contact with study authors to identify additional studies) in the search and date last searched.	9
Search	8	Present full electronic search strategy for at least one database, including any limits used, such that it could be repeated.	9
Study selection	9	State the process for selecting studies (i.e., screening, eligibility, included in systematic review, and, if applicable, included in the meta-analysis).	9-10
Data collection process	10	Describe method of data extraction from reports (e.g., piloted forms, independently, in duplicate) and any processes for obtaining and confirming data from investigators.	10
Data items	11	List and define all variables for which data were sought (e.g., PICOS, funding sources) and any assumptions and simplifications made.	NA
Risk of bias in individual studies	12	Describe methods used for assessing risk of bias of individual studies (including specification of whether this was done at the study or outcome level), and how this information is to be used in any data synthesis.	10
Summary measures	13	State the principal summary measures (e.g., risk ratio, difference in means).	11
Synthesis of results	14	Describe the methods of handling data and combining results of studies, if done, including measures of consistency (e.g., I^2) for each meta-analysis.	11

For peer review only - http://bmjopen.bmj.com/site/about/guidelines.xhtml

PRISMA 2009 Checklist

Section/topic	#	Checklist item	Reported on page #
Risk of bias across studies	15	Specify any assessment of risk of bias that may affect the cumulative evidence (e.g., publication bias, selective reporting within studies).	11
Additional analyses	16	Describe methods of additional analyses (e.g., sensitivity or subgroup analyses, meta-regression), if done, indicating which were pre-specified.	NA
**RESULTS**			
Study selection	17	Give numbers of studies screened, assessed for eligibility, and included in the review, with reasons for exclusions at each stage, ideally with a flow diagram.	12
Study characteristics	18	For each study, present characteristics for which data were extracted (e.g., study size, PICOS, follow-up period) and provide the citations.	16-18
Risk of bias within studies	19	Present data on risk of bias of each study and, if available, any outcome level assessment (see item 12).	17
Results of individual studies	20	For all outcomes considered (benefits or harms), present, for each study: (a) simple summary data for each intervention group (b) effect estimates and confidence intervals, ideally with a forest plot.	14-17
Synthesis of results	21	Present results of each meta-analysis done, including confidence intervals and measures of consistency.	17
Risk of bias across studies	22	Present results of any assessment of risk of bias across studies (see Item 15).	17
Additional analysis	23	Give results of additional analyses, if done (e.g., sensitivity or subgroup analyses, meta-regression [see Item 16]).	NA
**DISCUSSION**			
Summary of evidence	24	Summarize the main findings including the strength of evidence for each main outcome; consider their relevance to key groups (e.g., healthcare providers, users, and policy makers).	19-20
Limitations	25	Discuss limitations at study and outcome level (e.g., risk of bias), and at review-level (e.g., incomplete retrieval of identified research, reporting bias).	21
Conclusions	26	Provide a general interpretation of the results in the context of other evidence, and implications for future research.	22
**FUNDING**			
Funding	27	Describe sources of funding for the systematic review and other support (e.g., supply of data); role of funders for the systematic review.	23

From: Moher D, Liberati A, Tetzlaff J, Altman DG, The PRISMA Group (2009). Preferred Reporting Items for Systematic Reviews and Meta-Analyses: The PRISMA Statement. PLoS Med 6(7): e1000097. doi:10.1371/journal.pmed1000097

For more information, visit: **www.prisma-statement.org**.

For peer review only - http://bmjopen.bmj.com/site/about/guidelines.xhtml
